# Mesenteric Panniculitis: An Unusual Presentation of Abdominal Pain

**DOI:** 10.7759/cureus.5100

**Published:** 2019-07-08

**Authors:** Ankit Patel, Yazan Alkawaleet, Mark Young, Chakradhar Reddy

**Affiliations:** 1 Miscellaneous, Quillen College of Medicine, East Tennessee State University, Johnson CIty, USA; 2 Internal Medicine, Quillen College of Medicine, East Tennessee State University, Johnson City, USA; 3 Gastroenterology, Quillen College of Medicine, East Tennessee State University, Johnson City, USA

**Keywords:** mesenteric panniculitis, sclerosing mesenteritis, abdominal pain

## Abstract

Sclerosing mesenteritis is a rare autoimmune disease that eventually evolves into fibrotic changes that usually affect the adipose tissue around the mesenteric vessels. It can present through a myriad of gastroenterological as well as constitutional symptoms, including but not limited to abdominal pain, diarrhea, fever, nausea, or vomiting. Although the exact etiology of the disease is yet to be determined, there are several predisposing factors, the most common of which is a previous history of abdominal trauma and/or surgery. Several case series have reported the association of sclerosing mesenteritis with prior abdominal surgery ranging from as low as 24% to as high as 53%.

## Introduction

Sclerosing mesenteritis is a rare autoimmune disease that eventually evolves into fibrotic changes that usually affect the adipose tissue around mesenteric vessels. It can present through a myriad of gastroenterological as well as constitutional symptoms, including but not limited to abdominal pain, diarrhea, fever, nausea, or vomiting [1]. Although the exact etiology of the disease is yet to be determined, there are several predisposing factors, the most common of which is a previous history of abdominal trauma and/or surgery. Several case series have reported the association of sclerosing mesenteritis with prior abdominal surgery, ranging from as low as 24% to as high as 53% [2-4].

## Case presentation

The patient was a 53-year-old male with a past medical history of hypertension, who presented with a two-week history of abdominal pain, colicky in nature, intolerance to food and liquids, radiating to the flanks on both sides, with no alleviating or relieving factors. One year before, the patient had cholecystectomy due to biliary dyskinesia. His hospital stay at that time was complicated by the development of a biliary leak treated with biliary stenting. He later also developed multiloculated abscess collection in the gallbladder fossa that was managed with external drainage and intravenous antibiotics.

Investigation

**Figure 1:**
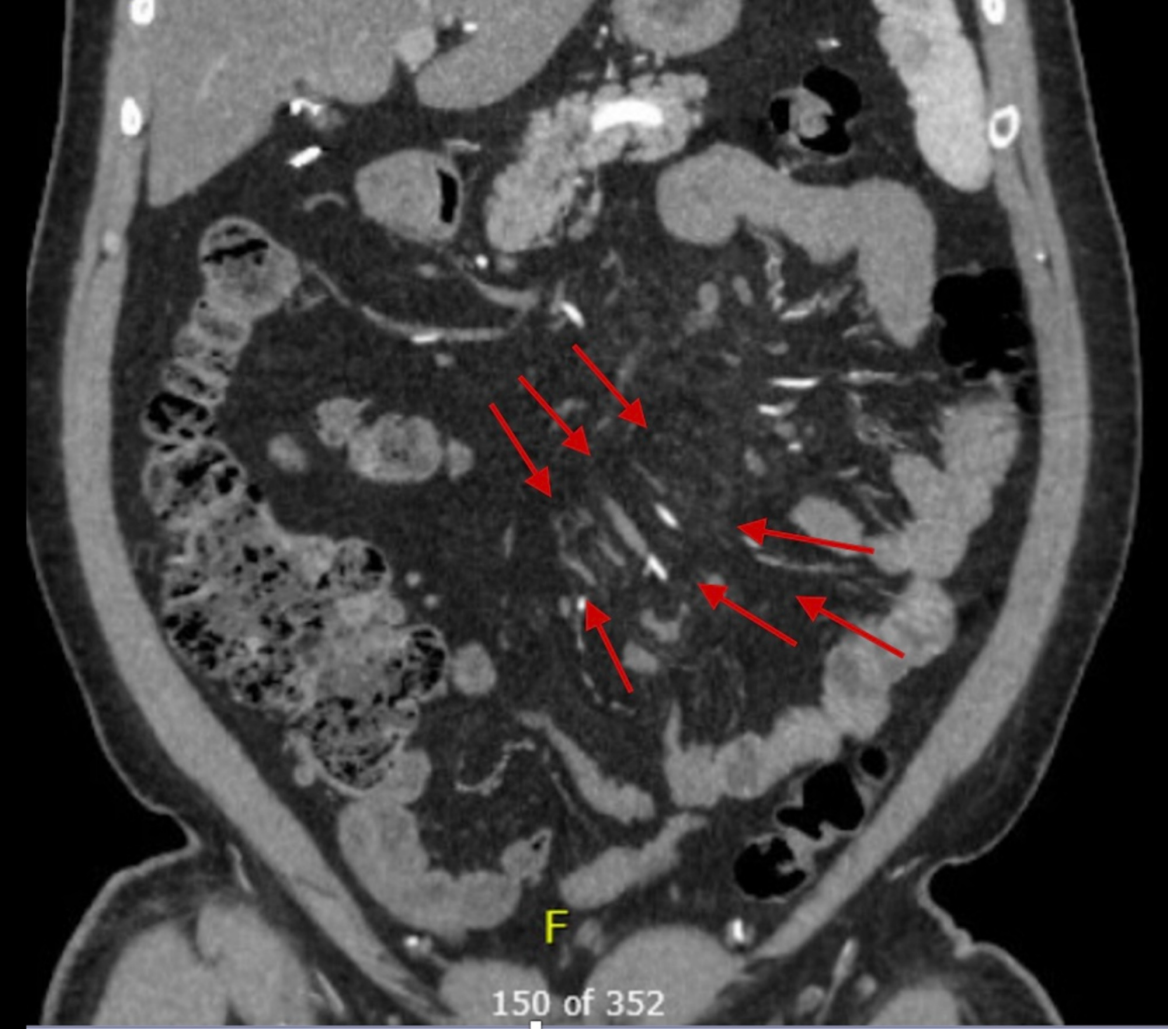
Computed Tomography of the Abdomen The red arrows denote the areas of mesenteric fat stranding.

**Figure 2:**
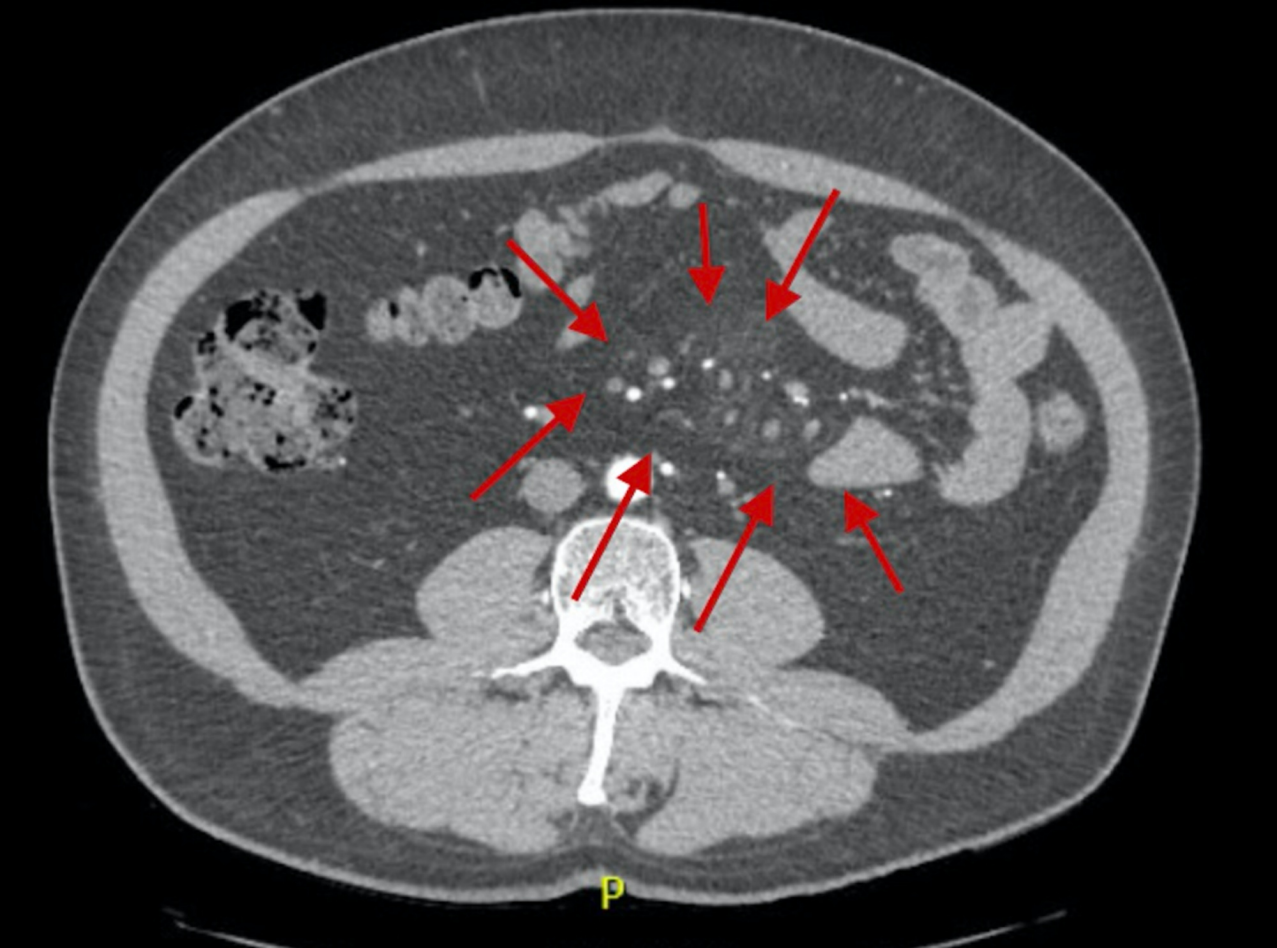
Computed Tomography of the Abdomen The red arrows denote the mesenteric fat stranding.

 In the emergency room, his vital signs were as follows: blood pressure from 120-130/70-80 mmHg, heart rate 70 beats per minute, temperature 97°F, and oxygen saturation more than 95%. On physical exam, he had abdominal tenderness in all four quadrants; no rigidity, rebound tenderness, masses, or skin changes were appreciated. Complete blood count showed a white blood count of 7000 µL with no shift to the left, hemoglobin of 15.6 g/dL and platelets of 107,000 µL. The complete metabolic panel showed sodium of 142 mmol/L, potassium 4.2 mmol/L, chloride of 19 mmol/L, glucose 99 mmol/L, calcium 9.5 mmol/L, lactic acid 1 mg/dL, lipase 50 U/L, phosphate 2.7, beta-hydroxybutyrate 0.12, and magnesium 1.9 mmol/L. Urine analysis and troponins were within normal limits. The electrocardiogram showed sinus rhythm. The urine drug screen was negative. C-reactive protein (CRP) and erythrocyte sedimentation rate (ESR) were within normal limits. Porphyria workup was negative. Alpha-1-antitrypsin was 123 mmol/L. The ceruloplasmin level was 17 mg/dL. Actin antibody, as well as the mitochondria M2 antibody, was negative. A computed tomography (CT) angiogram of the abdomen showed patent mesenteric vessels. However, fat stranding was noticed, especially at the root of the mesenteric vessels (Figures 1-2).

Treatment

 The patient was on prednisone 40 mg qday and tamoxifen. His abdominal pain was greatly improved after two days. He was discharged with prolonged prednisone taper. He was scheduled for an appointment with his primary care at discharge but didn’t show up and was lost to follow-up.

## Discussion

Terminology

Sclerosing mesenteritis is a coverage term that describes any inflammatory or sclerosing process of the abdominal mesentery. The disease has been referred to as mesenteric panniculitis, mesenteric lipodystrophy, as well as some other names. This case will use mesenteric panniculitis (MP) in reference to the disease.

Epidemiology

MP has been shown to be a rare finding and disease. Daskalogiannaji et al. showed that in 7000 computed tomography studies, the prevalence was 0.6% [1]. MP typically occurs in the fifth decade of life although a case as young as three years old have been reported. The reason many believe MP occurs in older individuals is that mesenteric fat increases with age. Studies have shown that MP is greater in males [2].

Etiologies and pathogenesis

The cause of MP is still debated and remains unclear. Some mechanisms have been suggested. The most common cause is abdominal surgery, which is seen in our patient who had a history of cholecystectomy.

Abdominal Surgery and Trauma

A systemic review of published literature showed that about 30% of patients have prior abdominal surgery [2]. A case review series of 92 patients showed that 41% had prior surgery, including cholecystectomy [3].

Autoimmune

This is postulated because MP has been rarely found as the initial presentation. Many times, it has presented with multifocal fibrosclerosis, limited systemic sclerosis, and celiac disease [3]. Autoimmune is also supported because MP responds to immunosuppressive therapy such as corticosteroids, azathioprine, and cyclophosphamide [2].

MP has been hypothesized to be an immunoglobulin G4 (IgG4)-mediated disease because of the abundance of IgG4 found on pathology [2,5].

Clinical manifestations

The symptoms of and signs of mesenteric panniculitis can vary. The disease can manifest as weight loss (20-23%), abdominal pain (30% to 70%), bowel movement changes such as diarrhea (25%) or obstruction [3,6-7]. The symptoms can last from days to years. About 10% to 15% are asymptomatic [1].

Lab values

Studies have shown that there are no specific laboratory values that can help differentiate mesenteric panniculitis [8]. One study has shown that 80% of patients have elevated ESR and CRP [8]. Our patient’s hematological studies, immunological studies, and chemistry panel came back within normal limits or negative.

Diagnosis

General Approach

MP is a rare disease that is often discovered incidentally. In a patient with no significant lab findings and obscure abdominal symptoms, CT can help with diagnosis. However, a biopsy via laparotomy or laparoscopically is needed to confirm the diagnosis.

Diagnostic Imagining

CT findings can vary, but the fat ring sign and tumor pseudocapsule help distinguish MP from lymphoma or carcinomatous and carcinoid tumors [8].

In mesenteric panniculitis, a soft tissue mass develops, which can envelope mesenteric vessels, leading to the development of collateral vessels. If surrounding fat is preserved, the fat ring sign can appear [8]. A tumor pseudocapsule is the outer band around the mesentery with soft tissue attenuation that protects normal tissue from the inflammatory process. The fat ring sign and tumor pseudocapsule are considered specific to mesenteric panniculitis, with Sabate et al. finding the tumor pseudocapsule in 50% of patients [9].

Treatment

No clear treatment has been established for mesenteric panniculitis. The current indication for treatment is to treat when the patient is symptomatic. If the patent is asymptomatic, they are likely to stay asymptomatic. The mainstay treatment is to provide therapy for the complications of mesenteric panniculitis. Typically, a corticosteroid taper and tamoxifen are used, as the case with our patient.

*Surgical Management* 

Obstruction treatment should stay conservative whenever possible. A surgical bypass should be considered where there are distinct spots of obstruction. However, a surgical bypass is often not feasible due to the blockage of mesenteric blood supply. Surgical treatment alone has not been proven to treat sclerosing panniculitis. In a case series by Akram et al., only 10% of patients that underwent surgery needed no additional treatment [3]. The most common indication for surgery is a severe bowel obstruction.

Medical Management

The majority of medical management is based on the use of corticosteroids [2]. Prednisone 40 mg and tamoxifen have been considered the mainstay therapy. If symptoms improved after three months, prednisone is tapered and tamoxifen is continued indefinitely. A number of immunomodulation agents have been tried such as cyclophosphamide or colchicine.

Prognosis

The Mayo model has incorporated many factors that can calculate the survival rate of mesenteric panniculitis (Table 1). The components of the Mayo risk score include age, serum bilirubin, serum albumin, serum aspartate aminotransferase (AST), and history of variceal bleeding. The calculation correlates well with overall survival rates [10]. Our patient did not have serum bilirubin and serum albumin lab values obtained before he was lost to follow-up, so his Mayo model score could not be calculated.

**Table 1:** Mayo model for predicted survival in primary sclerosing cholangitis [10]

R = 0.03 (age [yrs]) + 0.54e(bilirubin [mg/dL]) + 0.54 loge(AST [IU/L]) + 1.24 (variceal bleeding [0=no/1=yes]) - 0.84 (albumin [g/dL])]
Survival function coefficient [S0(t)]
1 year = 0.963
2 years = 0.919
3 years = 0.873
4 years = 0.833
Calculated patient survival
Probability of survival at time t years is calculated as S(t)= S0(t)exp(R-1.00)

## Conclusions

Mesenteric panniculitis is a rare cause of abdominal pain, and diagnosis is usually rarely inferred from the clinical presentation. Diagnosis is often suggested by radiological features; however, histological proof is essential for a definitive diagnosis. There is no consensus on the optimal treatment option, but prolonged steroid taper has been used, with various degrees of success.
